# Papillary fibroelastoma of the papillary muscle—a case report on a rare entity and sometimes a delayed diagnosis

**DOI:** 10.1093/ehjcr/ytaf529

**Published:** 2025-10-14

**Authors:** Jules Miazza, Oliver Reuthebuch, Thierry Carrel

**Affiliations:** Department of Cardiac Surgery, University Hospital Basel, Petersgraben 4, 4031 Basel, Switzerland; Department of Cardiac Surgery, Inselspital Bern, University Hospital Bern, Freiburgstrasse 20, 3010 Bern, Switzerland; Department of Cardiac Surgery, University Hospital Basel, Petersgraben 4, 4031 Basel, Switzerland; Department of Cardiac Surgery, University Hospital Basel, Petersgraben 4, 4031 Basel, Switzerland; CardioBern AG, Lindenhofspital, Bremgartenstrasse 117, 3001 Bern, Switzerland

**Keywords:** Cardiac surgery, Cardiac tumour, Papillary fibroelastoma, Case report

## Abstract

**Background:**

Papillary fibroelastoma, generally affecting the valve endocardium, is one of the most common benign cardiac tumours. Although rarely reported, atypical localization of such tumours complexifies diagnosis and might negatively impact patient outcomes due to late detection.

**Case summary:**

We present the case of a 69-year-old female patient presenting for a check-up consultation and reporting symptoms of dysesthesia, aphasia, and dizziness. The patient had a history of atrial fibrillation on anticoagulation with rivaroxaban starting 6 years prior to presentation. Further examination using transthoracic and transoesophageal echocardiography revealed a hyperechogenic structure at the level of the subvalvular apparatus of the mitral valve. Interestingly, when cardiac MRI was subsequently performed, this diagnosis could not be confirmed. This discrepancy might be linked to the lower spatial and temporal resolution compared to transoesophageal echocardiography, or transthoracic echocardiography with a high-frequency probe. To further investigate the symptoms of dysesthesia, aphasia, and dizziness, a cerebral MRI was performed which revealed multiple, bilateral ischaemic lesions. Considering the findings and the symptoms of the patient, the choice was made to perform explorative surgery. Intraoperatively, an 8-mm structure was identified and removed from the basis of the posteromedian papillary muscle. Histological examination revealed a papillary fibroelastoma. The patient was discharged on postoperative day 10. At follow-up, there was no report of neurological symptoms.

**Discussion:**

This case shows a highly atypical localization of a rare cardiac tumour and exemplifies the importance of multimodal diagnostic imaging prior to cardiac surgery.

Learning points

- In the diagnosis of an intracardiac tumour, multimodal diagnosis is essential. When atypically located, papillary fibroelastoma might not appear in cardiac MRI.
- In the era of improved non-invasive diagnostics, surgical exploration remains an option in well-selected cases.

## Introduction

Papillary fibroelastoma, one of the most common cardiac tumours, primarily affects the aortic and mitral valve.^1^ Although typically linked to valve endocardium, its localization can exceptionally differ. Considering the potential dramatic complications, such as ischaemic stroke and myocardial infarction, early diagnosis and treatment of this entity are of paramount importance.

## Summary figure

Created in https://BioRender.com

**Figure:**
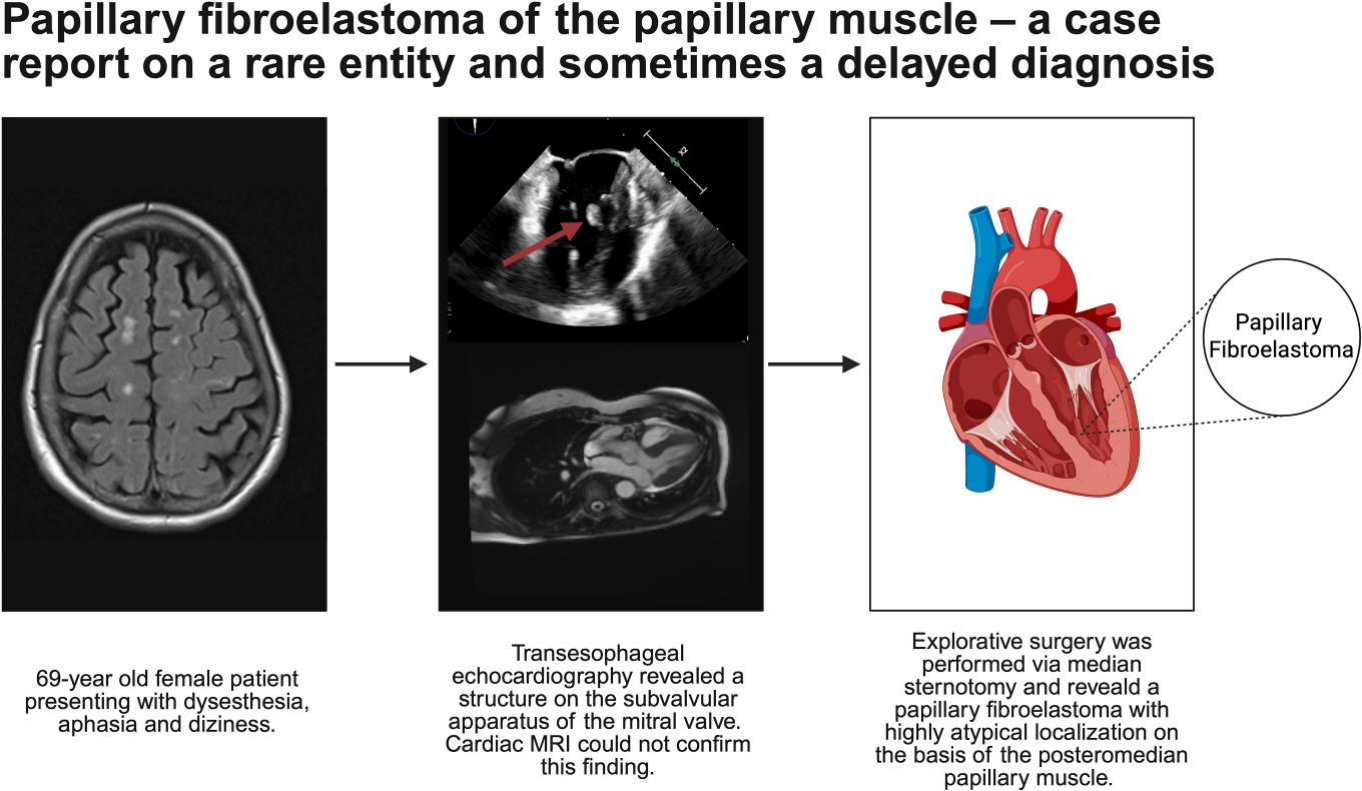


## Case presentation

**Figure 1:**
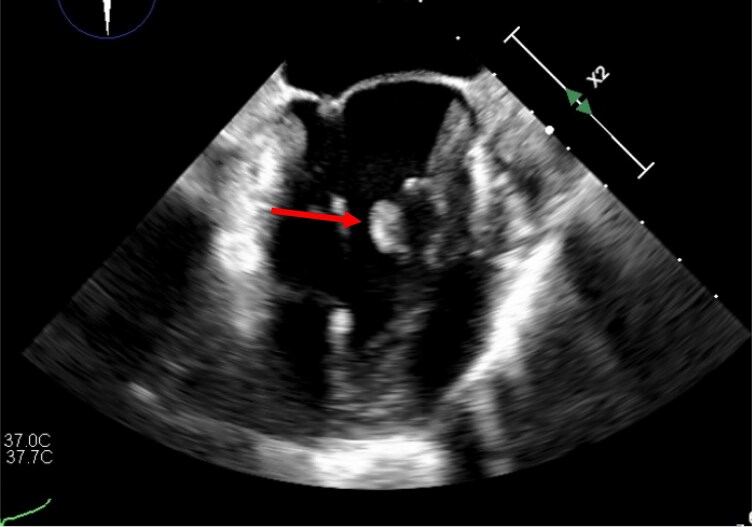
Transoesophageal echocardiography showing a mobile intracardiac mass on the posterior papillary muscle (arrow).

**Figure 2:**
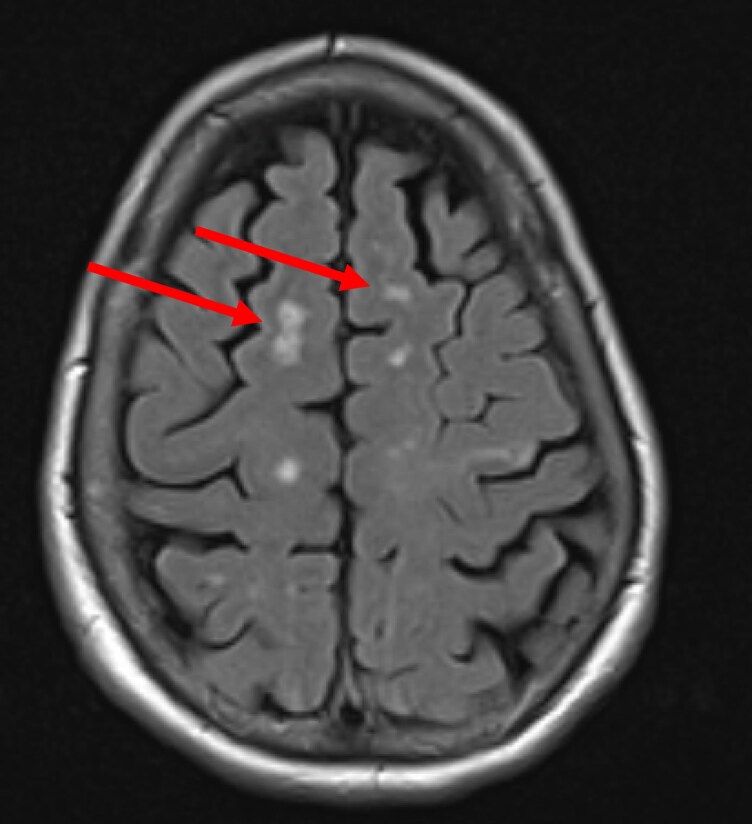
Brain MRI showing multiple bilateral lacunar stroke (arrow).

We present the case of a 69-year-old female patient presenting for a follow-up cardiology consultation in the context of atrial fibrillation. Shortly before the presentation, the patient experienced an episode of dysesthesia, aphasia, and dizziness. These symptoms were initially attributed to an embolism related to the arrhythmia, even though the patient was anticoagulated with Rivaroxaban 20 mg/d. Considering the normal renal function and body mass index, this dosage was considered adequate, but etiologic investigations were pursued. Physical examination demonstrated no neurological deficits. Cardiac and pulmonary auscultation findings were unremarkable. The echocardiography revealed a hyperechogenic structure at the level of the subvalvular apparatus of the mitral valve. In the retrospective analysis of an echocardiography performed 4 years prior, a 2-mm structure was already apparent at the same level. At this time, differential diagnosis included atypically located intracardiac tumours, intracardiac thrombus, and chordal rupture. Endocarditis was considered unlikely due to the absence of clinical features, laboratory abnormalities, risk factors, or recent exposure. Cardiac MRI was used to further investigate this finding and complete the diagnostic work-up. Interestingly, the MRI revealed no cardiac anomaly, especially no intracardiac mass. The patient was declined for surgery by another surgical team. Finally, transoesophageal echocardiography confirmed the presence of a 0.7 × 1.2 cm structure on the medial part of the anterolateral papillary muscle (*Figure 1*). To investigate the neurological symptoms, a brain MRI was performed. It revealed multiple, bilateral ischaemic lesions (*Figure 2*), potentially correlating with multiple embolic episodes from the intracardiac structure.

**Figure 3:**
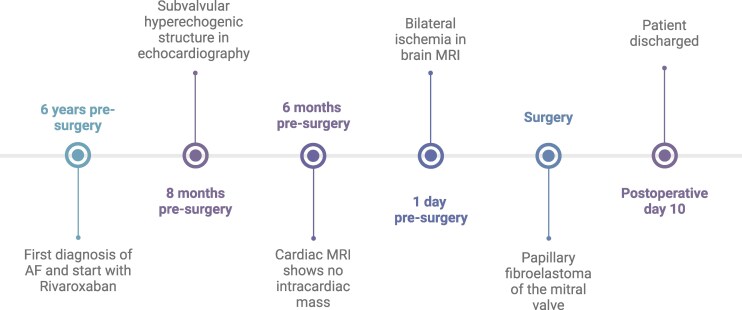
Timeline. Created in https://BioRender.com

In light of this diagnosis, the conservative and operative options were evaluated and discussed with the patient. Considering that the ischaemic stroke occurred while on anticoagulation and that the patient fortunately had no sequelae and maintained excellent quality of life, removing the mass and performing cryoablation and left atrial appendage closure appeared as a viable approach to reduce the risk of further strokes. The patient shared the opinion of the treatment team and expressed the wish for an operative approach, a decision supported by the 2022 ESC Guidelines on cardio-oncology of the European Hematology Association (EHA), the European Society for Therapeutic Radiology and Oncology (ESTRO), and the International Cardio-Oncology Society (IC-OS).^2^ To increase perioperative safety, a brain MRI was repeated the day before surgery to exclude any new haemorrhagic transformation. On the day prior to surgery, laboratory investigations revealed a haemoglobin level of 142 g/L (reference range: 120–160 g/L), platelet count of 252 × 10⁹/L (reference range: 150–450 × 10⁹/L), and a C-reactive protein concentration of 0.4 mg/L (reference value: < 10 mg/L). We opted for median sternotomy to offer optimal visualization, avoid fragmentation of the mass, and be able to treat any unexpected findings. Through a left atriotomy, a gelatinous structure was identified, lying between the base and head of the posteromedian papillary muscle, deep into the left ventricle. The tumour was entirely resected and, to avoid recurrence, a tangential resection of the papillary muscle at the site of tumour attachment was performed. The mitral valve was inspected and showed no lesions. Subsequently, we performed cryoablation and left atrium appendage closure from inside the atrium with a running suture. Intraoperative evolution was uneventful, but the ICU course was complicated by fluid overload, leading to significant tricuspid regurgitation, which was treated conservatively. The patient was transferred to the cardiac surgery ward on postoperative day 4 and discharged on postoperative day 10 after an uneventful stay. The histological examination of the excised mass revealed a papillary fibroelastoma and confirmed the completeness of the resection. At the 3-months follow-up, the patient had recovered and reported no further neurological symptoms. *Figure 3* depicts the process from diagnosis to discharge with the help of a timeline.

## Discussion

Papillary fibroelastoma of the heart normally originates from the valvular endocardium.^1^ In our case, the tumour was situated directly behind the papillary muscle on the ventricular side. Such atypical localizations of papillary fibroelastoma have been described in the literature, but usually closer to the chordae tendinae.^3–6^ Possibly due to this uncommon location, small size, and hypermobility, the mass was not visualized by cardiac MRI. In such cases, the use of transoesophageal echocardiography is unrivalled. This case highlights the importance of multimodal diagnosis in an interdisciplinary setting to offer patient-tailored therapeutic options and to prevent recurrent cerebral embolization.

## Conclusion

Papillary fibroelastoma can present a diagnostic challenge, particularly when located on the papillary muscles. Multimodal imaging is essential to establish an accurate diagnosis and to guide a patient-tailored therapeutic approach.

## Lead author biography


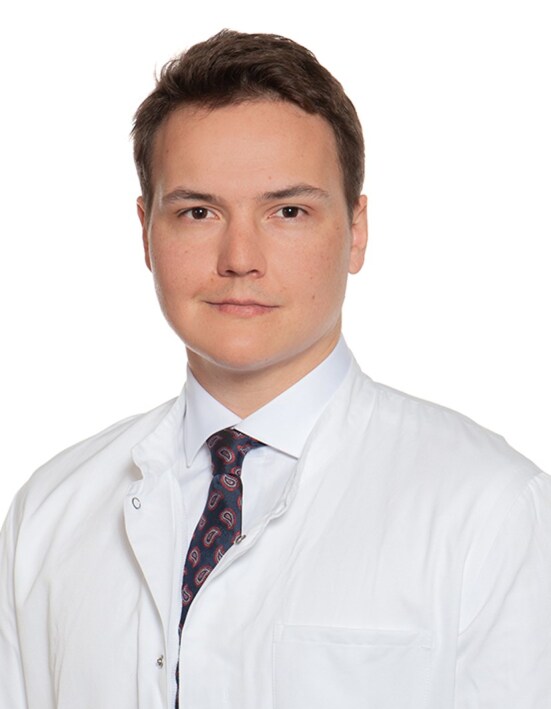


Dr Jules Miazza is a cardiac surgery resident from University Hospital Bern, Switzerland. Dr Miazza’s clinical interests are aortic surgery, minimally invasive surgery, and hybrid procedures. His scientific interests include aortic surgery and aortic imaging, and the use of artificial intelligence in cardiac surgery.

## Supplementary Material

ytaf529_Supplementary_Data

## Data Availability

The data underlying this article will be shared on reasonable request to the corresponding author.
